# The Transport of Microplastics from Soil in Response to Surface Runoff and Splash Erosion

**DOI:** 10.1021/acs.est.5c04795

**Published:** 2025-07-02

**Authors:** Emilee Severe, Ben W. J. Surridge, Peter Fiener, Michael P. Coogan, Rachel H. Platel, Mike R. James, John Quinton

**Affiliations:** † Lancaster Environment Centre, 4396 Lancaster University, Lancaster LA1 4YQ, U.K.; ‡ Institute of Geography, University of Augsburg, Alter Postweg 118, Augsburg 86159, Germany; § Department of Chemistry, Lancaster University, Lancaster LA1 4YB, U.K.

**Keywords:** microplastics, surface runoff, splash erosion, soil erosion, environmental pollution, fluorescence

## Abstract

Erosion is hypothesized to be a significant process transporting microplastics (MPs) from soils to aquatic environments, however, the factors controlling this process are poorly understood. Using a novel combination of high-frequency photography and fluorescent particles, we compared the transport of three MPs to that of a sand particle during rainfall simulations: linear low-density polyethylene (LLDPE), polystyrene (PS), and poly­(methyl methacrylate) (PMMA). We measured the “real time” movement of particles on the soil surface alongside the number of particles transported through splash erosion and surface runoff. Our results show that MPs of all polymer types demonstrated more rapid transport from the soil surface compared to sand particles throughout the rainfall simulations. Prior to surface runoff, ∼65–75% of MPs and sand particles were removed from the soil surface through raindrop-driven incorporation into the soil matrix. Surface runoff and splash erosion accounted for the transport of approximately 47% of PMMA and 57% of PS, while only 30% of sand particles were mobilized by these processes. This research establishes a benchmark for evaluating MP mobility to current knowledge of soil particle movement, which is critical for estimating the redistribution of MPs within soils and their ultimate flux to aquatic ecosystems.

## Graphical abstract

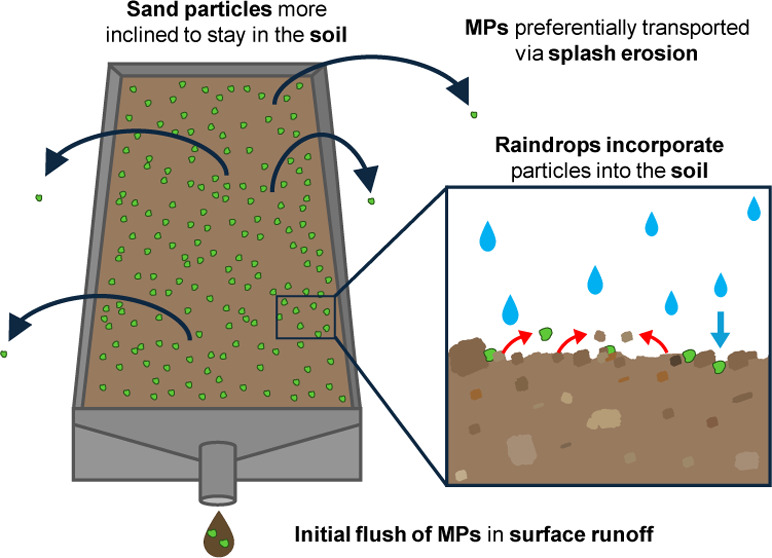

## Introduction

Microplastics (MPs) are emerging contaminants of concern and are increasingly recognized as persistent pollutants with the capacity to significantly disrupt the ecological function of Earth’s systems.^1,2^ Plastic products are produced and primarily used in the terrestrial environment, with few exceptions such as within the fishing industry. Nevertheless, MPs have been detected in abundance within a variety of ecosystems across the globe, spanning terrestrial,^3–5^ aquatic,^6,7^ polar^8–10^ and anthropogenic ecosystems.^11–13^ In particular, agricultural soils, have been identified as a potentially major sink for MPs, partly due to the accumulation of MPs from a multitude of input sources including fragmentation of agricultural plastic products, biosolids, compost, road runoff and atmospheric deposition.^14–20^ While multiple processes drive the global redistribution of MPs, the processes governing the transport of MPs from terrestrial environments to aquatic ecosystems remain particularly poorly researched.

Erosion processes, such as surface runoff and splash erosion, are well-documented mechanisms governing the detachment and transport of soil particles and agricultural pollutants from soils to aquatic ecosystems.^21–24^ Research to date which examines MP transport in surface runoff has largely focused on how variations in MP characteristics, such as size and morphology, or soil conditions influence MP transport.^25–28^ However, critical gaps exist in our understanding of the mechanisms of MP movement in surface runoff and splash erosion processes, and in terms of how MP transport processes fundamentally differ from what is currently known about soil particle movement.

The movement of mineral soil particles in surface runoff and splash erosion processes have been long-studied, although MPs have a number of unique properties compared to organic and mineral soil particles which could facilitate distinct mobilization and transport behaviors.^29,30^ These properties include: (1) the relatively low density of MPs (∼1 g cm^–3^) compared to the average density of mineral soil particles (∼2.65 g cm^–3^),^31^ and (2) the specific physicochemical properties of MPs including hydrophobicity, plasticity and surface charge.

In this context, we sought to compare the movement of sand particles, a surrogate for mineral soil particles, to several types of MPs within the soil environment. Using fluorescent sand particles and MPs, we develop a highly novel approach to track in “real-time” the movement of particles during rainfall simulations, allowing us to quantify differences between the rate and pathways (surface runoff, vertical flux into the soil, and splash erosion) for transport of the MP and sand particles. We hypothesize that (1) MPs will show more rapid rates of movement from the soil surface compared to sand particles throughout the rainfall event and (2) MPs of all polymer types will be preferentially eroded both through surface runoff and splash erosion as compared to the sand particle, due to physical and chemical differences between the particle types, for example, in terms of density and hydrophobicity. Understanding the similarities and differences in the transport processes between MPs and mineral soil particles is critical not only for estimating MP fluxes from terrestrial to aquatic ecosystems, but also for evaluating the effectiveness of soil erosion research methodologies to understand MP transport dynamics and the capacity of erosion control practices to reduce MP loads entering aquatic ecosystems.

## Methods

### Experimental Set-Up

Metal soil boxes (width, length and depth of 24.5 cm × 50 cm × 10 cm) were packed with a naturally sourced loamy sand topsoil from Norfolk, UK which was screened to 4 mm (Bailey’s of Norfolk LTD). Particle size range distribution of the soil was 7.8 ± 1.7% clay; 7.6 ± 0.4% silt; 84.7 ± 1.9% sand and an organic matter content of 3%. Soil was added in five separate 2.2 cm layers, packing to a bulk density of 1.3 g cm^–3^. Soil volumetric water content was brought to 19% during the packing process by adding a known volume of tap water to each soil layer (see Supporting Information 1.1). Soil boxes were set at a 10-degree slope. A 1 m × 1 m wooden frame covered in black geotextile fabric was placed beside the soil box, to determine the quantity of particles transported out of the soil box through splash erosion (Figure S1). Rainfall was simulated using a gravity-fed rainfall simulator set at 50 mm h^–1^ with a Christiansen’s coefficient of 84.5%^32^ and a kinetic energy of approximately 14.59 J m^–2^ mm^–1^. Overall, a mean rainfall rate of 49 ± 2 mm h^–1^ was recorded (Figure S2). While this rainfall rate is considered an extreme rainfall event in the UK,^33,34^ high rainfall rates are commonly used in erosion experiments in order to rapidly saturate the soil and to induce surface runoff, thereby facilitating the study of erosion processes.^35–37^ Additional information about the soil properties, rainfall simulator and experimental setup can be found in Supporting Information Section 1.1.

### Particle Tracers

Four types of fluorescent particles were used in the research: linear low-density polyethylene (LLDPE); poly­(methyl methacrylate) (PMMA); polystyrene (PS); and sand. LLDPE was chosen as it is a common polymer used in agricultural mulch films,^38,39^ and while PS and PMMA are not common polymers used in agricultural products^39,40^ they have been detected in varying amounts in agricultural soil.^12,41^ Fluorescent PMMA (Simply Plastic Ltd.) and PS (Mark SG Enterprises Ltd.) were purchased commercially, and the polymer types were confirmed via FTIR. To create a fluorescent LLDPE particle, LLDPE (Sigma-Alrich) was fused to a homogeneous mixture with a modified lipophilic Rhodamine B derivative (S1.2) and characterized by fluorescence spectroscopy. The sand particles used were comprised of a sand core with a green fluorescent coating^42^ (Partrac Ltd.).

Density varied slightly between each of the particle types: LLDPE = 0.92 g cm^–3^, PMMA = 1.19 g cm^–3^, PS = 1.05 g cm^–3^ and the sand particles = 2.65 g cm^–3^. Particle morphologies were visually determined using charts based on Powers’^43^ particle shape classification system. The sand particle had a spherical subangular morphology. All plastic types were milled using a Cryomill (Verder-Scientific) which gave the PMMA and PS a spherical subangular morphology (Figure S3). LLDPE assumed a thinner, flake-like morphology after milling, which was subsequently classified as a nonspherical subrounded morphology.

Each particle type was dry sieved using an automated shaker (Endecotts Ltd.) to commonly detected size ranges in agricultural fields.^3,5,16,44,45^ LLDPE was sieved into two size ranges: small 250–355 μm (LLDPE_S_) and large 500–600 μm (LLDPE_L_). The PMMA, PS and sand particles were sieved to 250–355 μm size range. The LLDPE_S_ proved difficult to detect and resulted in poor recovery during our experiments, causing us to exclude it from the research reported here (see Supporting Information 1.3). A weight-to-particle number ratio was calculated for each particle type (Supporting Information 1.4), and approximately 10,000 particles of each particle type were spread evenly on the surface of the soil within each soil box immediately before the soil box was placed under the rainfall simulator. The input concentration of 10,000 particles was arbitrarily chosen to ensure sufficient particles would be detected, without compromising the ability to detect individual particles on the soil surface. Estimates from images of the soil surface after the initial input of particles suggested a mean particle count of 9781 ± 976. PS had the highest mean count of 10790 ± 347 particles, sand and PMMA had similar counts of 9590 ± 1381 and 9525 ± 814 particles, respectively, and LLDPE_L_ had the lowest mean count of 9219 ± 396 particles. Each particle type was placed in separate soil boxes, rather than combined particle treatments within individual soil boxes, and blank soil boxes without the addition of any particles were used to account for background fluorescence in the soil and reflection of UV light on the water during data collection (see below).

### Image Collection

Two cameras were used to capture images of both the particles moving over the soil surface and the particles transported out of the soil box via splash erosion during the rainfall simulations (Figure 1). A Canon EOS 850D camera with a 50 mm prime lens was used to capture images of the soil surface with an intervalometer (Neewer RS-60 × 10^3^) programmed to take images every 10 s. Camera settings were tested and optimized under UV lighting (Supporting Information 1.5). An additional camera, a Canon EOS 500D, recorded images of a 50 cm × 50 cm subarea of the 1 m × 1 m splash mat, marked with a metal quadrat, directly beside the soil box to record the number of MP and sand particles transported through splash erosion over time. As for the soil surface photography, a 50 mm prime lens was used, to photograph the area of the splash mat with an intervalometer (Neewer RS-60E3) programmed to capture images every 60 s. Prior to the start of the rainfall simulations, lenses on both cameras were autofocused with the laboratory room lights on, then switched to manual focus and the focus ring was manually secured to prevent focus drift due to shutter vibrations.^46^ At the conclusion of each simulation, the entire 1 m^2^ splash mat was photographed and used to calculate the total number of particles transported by splash erosion (Figure S4).

**Figure 1:**
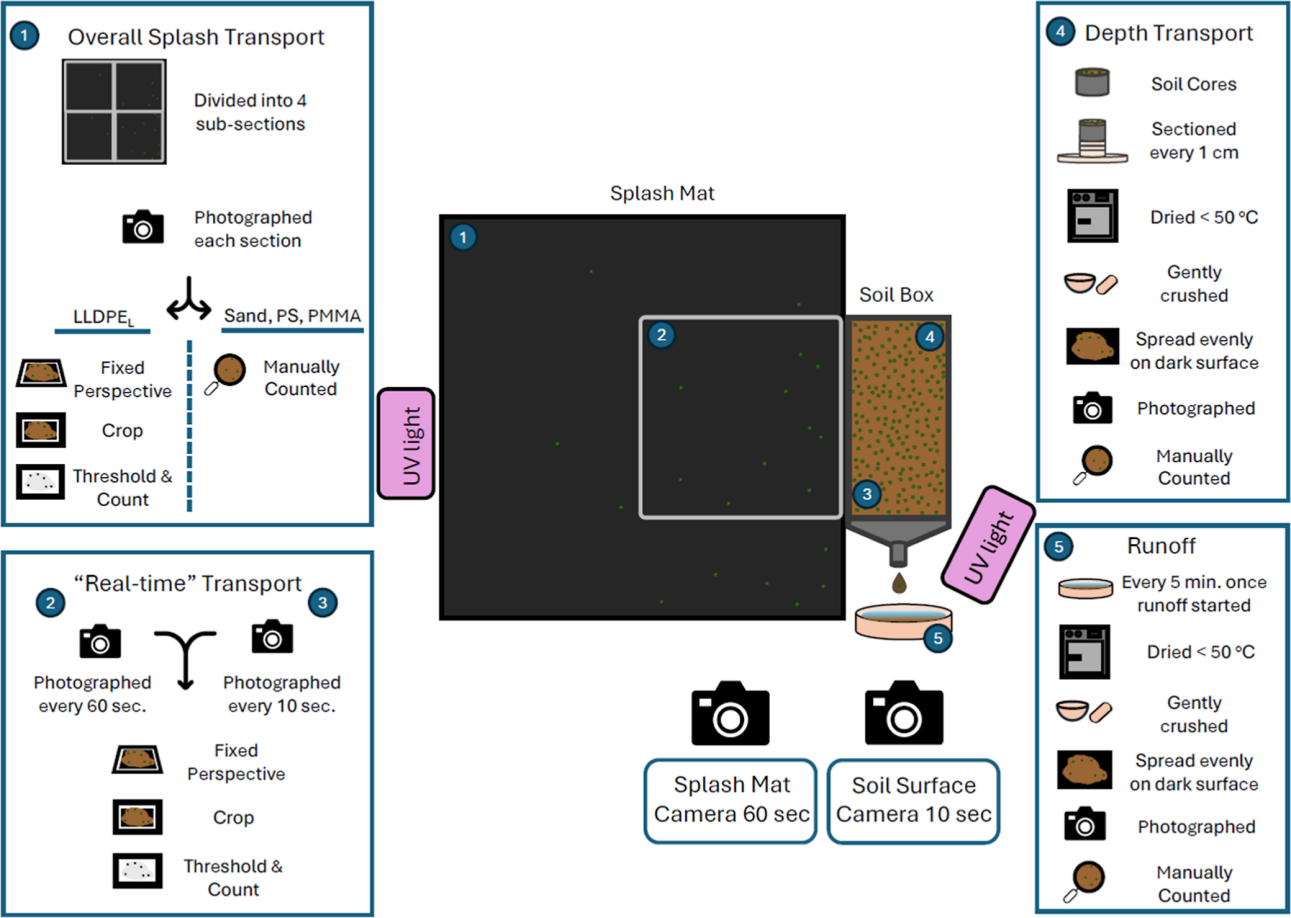
Diagram showing the experimental setup, sample collection and sample processing. LLDPE_L_; PMMA; PS; and SAND represents linear low-density polyethylene size large, poly­(methyl methacrylate), polystyrene and sand particles, respectively.

Two 50 W UV floodlights with a peak emission at 365 nm (Mark SG Enterprises) were used to excite the fluorescent dyes in the MP and sand particles.^47^ One floodlight was positioned to illuminate the soil box, and the other was positioned to illuminate the splash mat. Windows in the laboratory were blacked out to eliminate visible light from the room.

### Surface Runoff and Soil Samples

Once surface runoff began, the surface runoff leaving each soil box was subsampled every 5 min for 25 min. After collection, samples were immediately weighed before being placed in a drying cabinet (maximum temperature 50 °C) until dry, then reweighed to determine the volume of surface runoff and the mass of sediment transported from each soil box. Runoff sediment was subsequently spread evenly on a dark surface, photographed, then fluorescent MPs and sand particles were manually counted (Figure 1).

Four soil cores (diameter = 5.3 cm) were taken from each soil box following the rainfall simulation. The locations for core sampling were chosen using stratified random sampling to account for variations in MP and sand particles downslope across the soil box. Soil samples were taken to a depth of 4 cm and sectioned every centimeter. Soil samples were then dried, spread thinly on a dark surface, photographed and the fluorescent particles in the images were manually counted (Figure 1).

### Image Processing and Analysis

All images were captured in RAW format and converted to TIFF (LZW compression) format using Adobe Photoshop. As the cameras could not be located to observe the soil box and splash mat surfaces orthogonally due to rainfall, perspective effects varied the size of the ground area represented by individual pixels within images. The Perspective Warp tool (stretch function) in Adobe Photoshop was used to resample the images, correcting for perspective in the images.^48^ Reference scales were placed at the top and bottom of the soil box and splash mat to validate the resampling.

Using ImageJ, each image was cropped just inside the edges of the soil box which created approximately a 24.5 cm × 50 cm area in the images. Splash mat images were cropped just inside the edges of the subarea marked with a metal quadrat, creating an approximate 50 cm × 50 cm area in the images. Images were then processed through the Color Thresholding tool in ImageJ to quantify the particle counts of MP and sand in each image, using a HSB color space specific to each particle type (see Supporting Information 1.6). The Watershed Separation tool was then used to identify and separate potential adjoining particles.

Microplastic and sand particles within the 1 m × 1 m splash mat were manually counted, except for the LLDPE particles which were counted with ImageJ thresholding. This was due to a lower resolution of the splash mat images compared to the images taken of the surface runoff and soil samples. LLDPE particles had a slightly weaker fluorescence compared to the commercially purchased plastics with the chosen UV wavelength. Combined, these two factors led to analysis with ImageJ for the LLDPE images of the splash mat.

### Performance Evaluation

Because of the dynamic nature of the soil surface and fluorescent particles during the rainfall simulations, it was challenging to quantify the effectiveness of the image-based detection of particles on the soil surface and splash mat. Due to changes in the soil surface throughout the simulations and the onset of water flowing on the soil surface, it is possible that there were some fluorescent particles on the surface of the soil that remained undetected using the approach developed for our research.

To quantify the effectiveness of the image analysis process, fluorescent particles in the images of the soil boxes and splash mats before, during and after the rainfall simulation were manually counted and used as ground truth. Performance of the image analysis was evaluated by calculating the f-score for each particle type (Supporting Information 1.7). F-score, also known as the harmonic mean of recall and precision, is calculated on a scale of 0 to 1, with 1 reflecting the highest performance. F-score considers both the proportion of particles detected and the proportion of the detected particles which were correctly classified in the thresholding procedure. Overall, for the soil surface, all particles had a f-score >0.88 meaning over 88% of particles were correctly classified. The splash mat showed similarly high rates of detection for sand, PMMA and PS (f-score >0.97), with LLDPE_L_ showing the lowest f-score of 0.81.

Additionally, blank soils without any fluorescent particles added to the surface were used to assess the potential number of false positives in the images, thereby allowing us to assess potential overestimation of particles in the data. The average number of particles detected on the blank soil surfaces and splash mats was deemed to be negligible for all particle types; and they were an order of magnitude lower in comparison to the soil boxes which had received particle inputs (see Supporting Information 1.8)

### Data and Statistical Analysis

To estimate the total number of sand and MP particles transported outside of the soil box via splash erosion for the mass balance, particle counts from the 1 m^2^ splash mat were used to approximate the transport in a 1 m circular radius around the soil box. Due to the sloped soil surface, more particles were expected to accumulate downslope relative to the upslope direction. However, by averaging the number of particles across the entire 1 m^2^ splash mat this directional variability was incorporated into the estimation.

Statistical analysis was performed using R Statistical Software version 4.3.1.^49^ Histograms of the data were visually inspected and normality was tested using D’Agostino-Pearson’s K^2^ test to a 0.05 significance. Analysis of variance test (ANOVA) was used to test for differences between treatments when the data were normally distributed along with a Tukey’s posthoc test. When data were not normally distributed, Kruskal–Wallis tests were used along with a Wilcoxen rank-sum posthoc test. The holm method was used in the Wilcoxen posthoc tests to reduce the likelihood of type 1 errors. Data regarding the real-time movement of fluorescent particles from the soil surface were log-transformed and fit to a linear model. The coefficients noted as “B” were compared by using 95% confidence intervals. Residuals of the linear models were found to fit a normal distribution, both visually with Q–Q plots and with D’Agostino-Pearson’s K^2^ test to a 0.05 significance. Data are reported as mean ± standard deviation unless otherwise noted.

## Results and Discussion

### Using Photography to Track the Real-Time Movement of Fluorescent Particles

Previous research has utilized fluorescent particles to study the movement of MPs and soil particles during erosion events.^42,50,51^ However, the use of impervious surfaces in some research^50,51^ limits our understanding of particle transport during erosion events by excluding the potentially significant pathway of vertical transport into the soil profile.^52,53^ Our research builds upon this work, to not only track particle movement on the soil surface, but also to identify key transport pathways taken by particles during erosion events.

The dynamic nature of both the soil surface, including variations in surface roughness and the onset of surface runoff, and of the fluorescent particles during the rainfall simulations, makes it challenging to detect fluorescent particles on the soil surface using image-based detection methods.^42^ It is possible that there were particles on the surface of the soil that remained undetected using the approach we developed. However, we remain confident that the patterns of particle movement on the soil surface that are reported can be attributed to transport processes, and do not simply reflect artifacts of the detection method (Supporting Information 1.7).

The number of particles detected on the soil surface through time revealed two distinct phases of particle movement on the soil surface for both MP and sand particles (Figure 2). First, an initial period of exponential decline in particle number on the soil surface followed, second, by a more gradual, linear decrease in particle number. The transition between these two phases was linked to the onset of surface runoff reaching the end of the soil box, which ranged between 15 and 32 min, with a mean start time of 24 min and 15 s.

**Figure 2:**
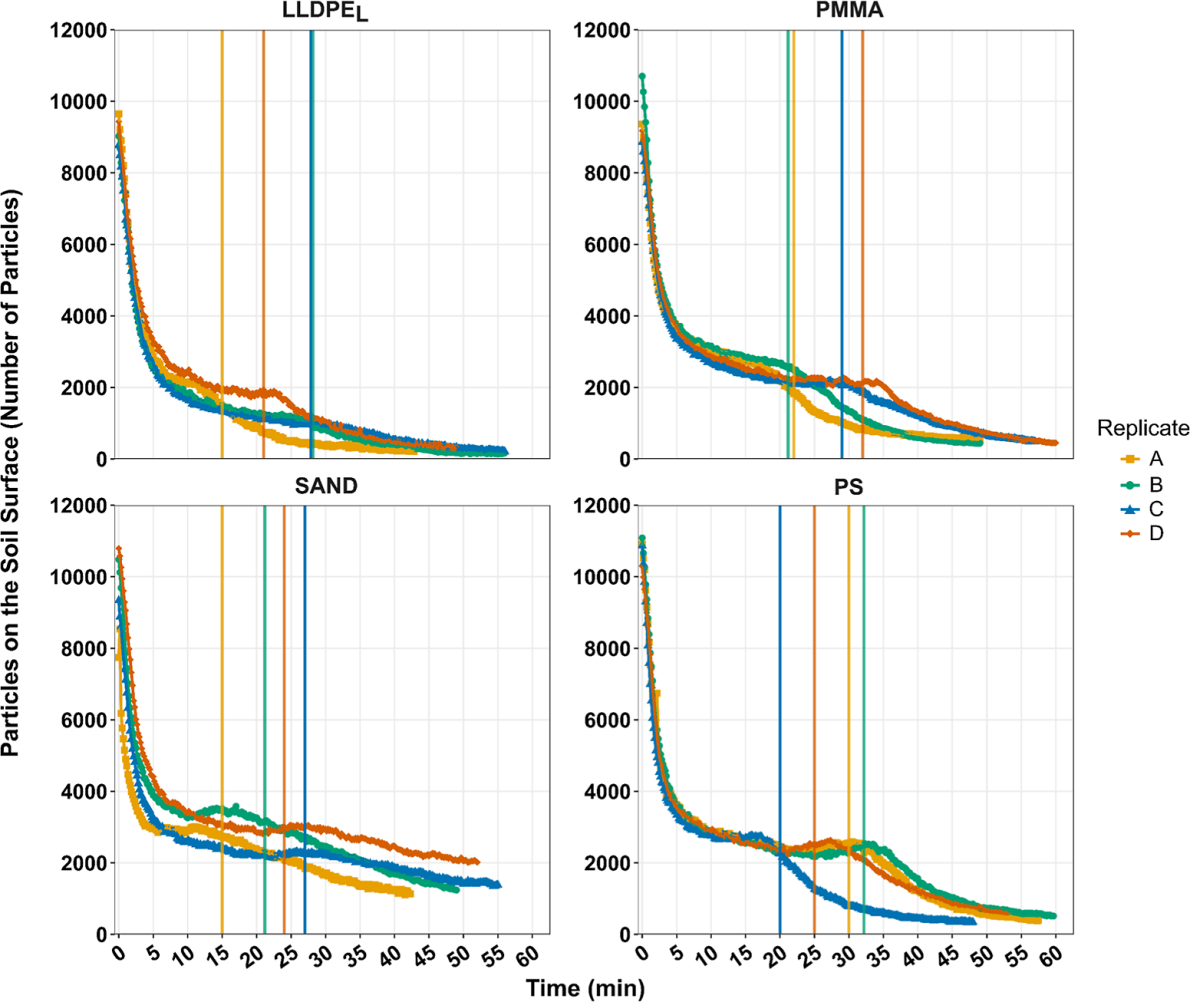
Number of particles on the soil surface through time. Vertical lines indicate the start of surface runoff delivery for each replicate. LLDPE_L_; PMMA; PS; and SAND represents linear low-density polyethylene size large, poly­(methyl methacrylate), polystyrene and sand particles, respectively.

To compare the rates of decrease in particle number detected on the soil surface between each particle type, data were split between the phases of movement prior to surface runoff and after surface runoff began, then log transformed and fit to linear models (Figure 3A,B). Slope coefficients from the linear models were compared between particle types and the lack of overlap in 95% confidence interval values were used as evidence of significant differences in slope coefficients. Prior to surface runoff initiation, LLDPE_L_ showed the most rapid rate of decline (*B* = −0.69), followed by PMMA, (*B* = −0.43) and PS (*B* = −0.42), and then sand (*B* = −0.37) (Figure 3A). All slope coefficients had a *p*-value <0.001. All particle types, apart from PMMA and PS had significant differences in slope coefficients (Figure 3A)

**Figure 3:**
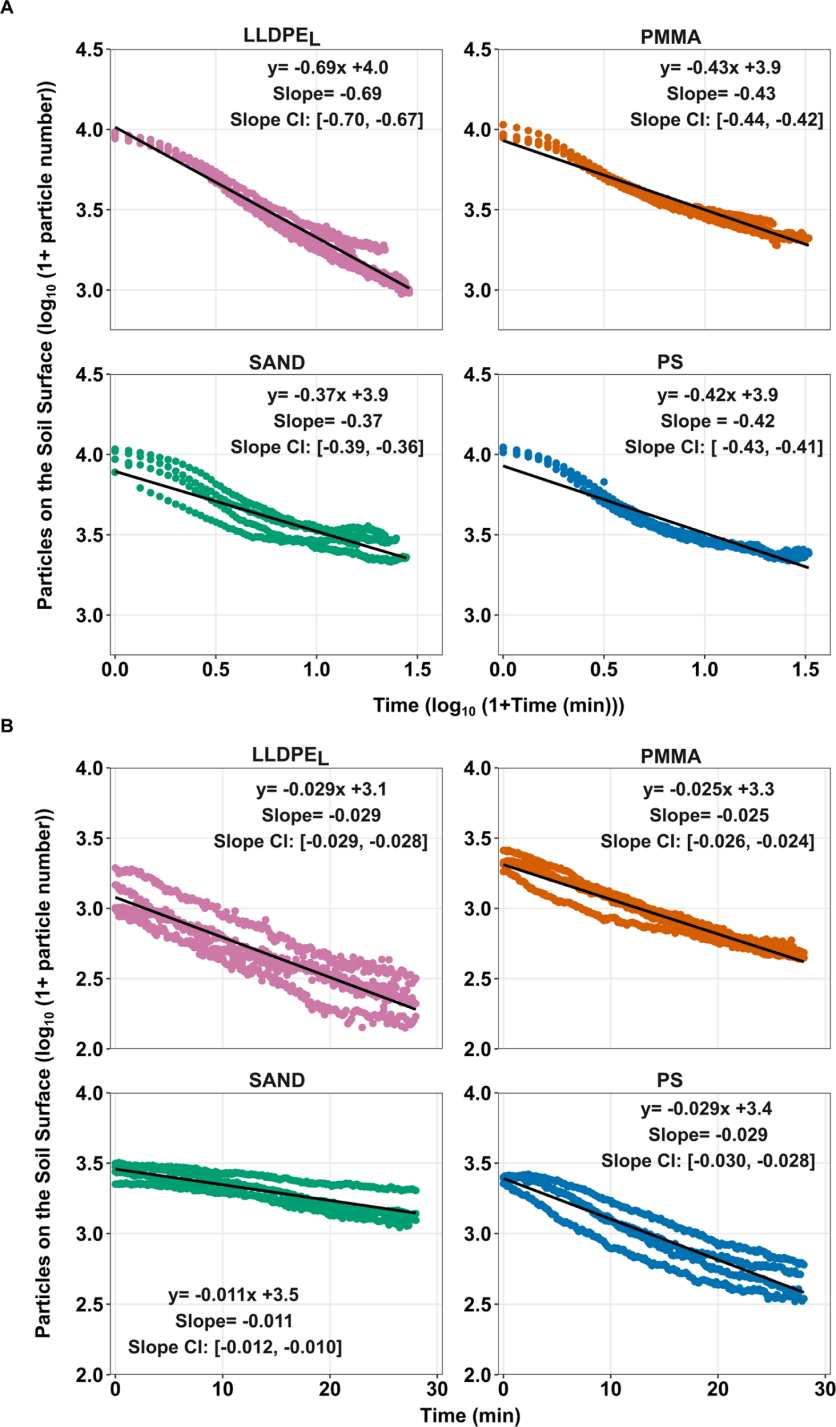
Log transformed data showing the rate of decline in number of particles from the soil surface. Panel A shows the decline in number of particles on the surface prior to surface runoff. Panel B shows the decline in numbers of particles from the surface after the onset of surface runoff. Data from four replicates are shown for each particle type in each panel. Empirical fit equations, slope coefficients and 95% confidence intervals for the slope coefficient are shown for each particle type. All slope coefficients had a *p*-value <0.001. Comparisons between the linear models in panel A and panel B should not be made due to the difference in *x*-axis. LLDPE_L_; PMMA; PS; and SAND represents linear low-density polyethylene size large, poly­(methyl methacrylate), polystyrene and sand particles, respectively.

A similar pattern was found after the onset of surface runoff (Figure 3B). All MP particle types: PS (*B* = −0.029; 95% CI [-0.030, −0.028]); LLDPE_L_ (*B* = −0.029; 95% CI [-0.029, −0.028]); and PMMA (*B* = −0.025; 95% CI [-0.026, −0.024]) were associated with faster rates of decline compared to the sand particles (*B* = −0.011; 95% CI [-0.012, −0.010].) All slope coefficients had a *p*-value <0.001.

The movement of particles by splash erosion differed significantly between particle types (Figure 4). The highest rate of particle accumulation on the splash mat was associated with PS (*B* = 12.40), followed by PMMA (*B* = 8.23), LLDPE_L_ (*B* = 4.62), and finally sand (*B* = 4.29). Each coefficient describing particle accumulation on the splash mats differed significantly between particle types, except for LLDPE_L_ and sand (95% CI: PS [11.89, 13.01]; PMMA [7.64, 8.82]; LLDPE_L_ [4.01, 5.23]; sand [3.65, 4.92].) For MPs, following the start of surface runoff, there was a slight increase in the rate of MP transport to the splash mat, a pattern which was not repeated for the sand particle.

**Figure 4:**
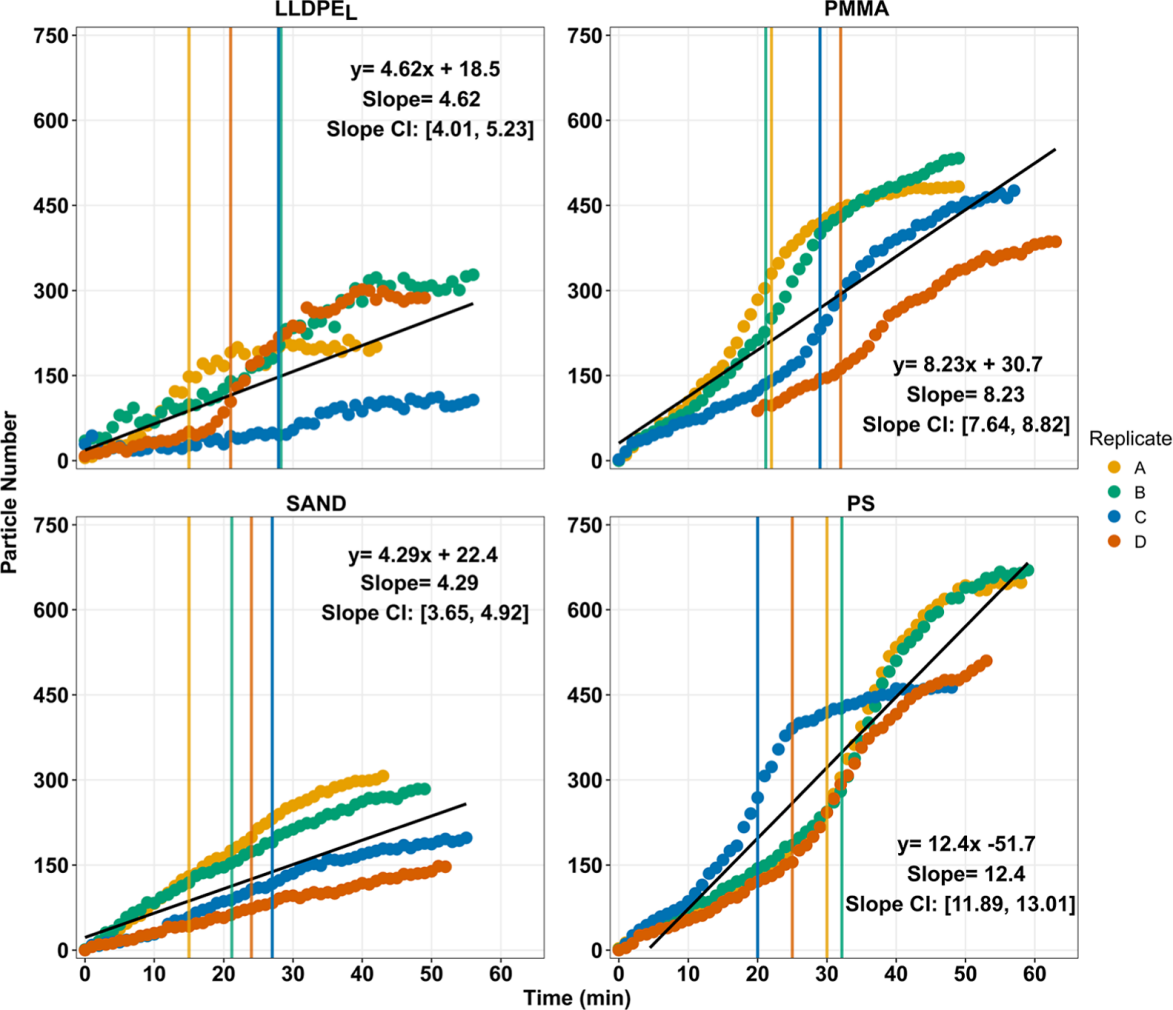
Number of particles on the 50 cm^2^ section of the splash mat through the full rainfall simulation. Each panel shows results from four replicates with each line representing a single replicate. Vertical lines indicate the start of surface runoff delivery for each replicate. For the MPs there is an increased rate of accumulation on splash mat when surface runoff begins; this pattern is absent for the sand particle. LLDPE_L_; PMMA; PS; and SAND represents linear low-density polyethylene size large, poly­(methyl methacrylate), polystyrene and sand particles, respectively.

Estimates of the number of particles transported by splash erosion (up to 50 cm radius from the edge of the soil box) were compared to the initial exponential decrease in particle number detected at the soil surface, to assess whether splash erosion could explain this decrease (Figures 2 and 4). A single time point at 600 s was used for this comparison, as this was prior to any surface runoff and therefore the reduction in particle number on the soil surface could not be attributed to surface runoff processes. Across all particle types, between 70 to 80% of particles (∼7000–8000 particles) disappeared from the surface of the soil box in the first 600 s of the rainfall simulation. Because only 3 to 5% of MP and sand particles (∼300–500 particles) were estimated to be transported out of the soil box by splash erosion during this period, splash erosion cannot explain the exponential reduction in MP and sand particle number on the soil surface reported above.

The primary hypothesis for the reduction in MP and sand particle number on the soil surface is raindrop impact incorporating particles into the soil. Raindrops can transform the surface of a soil by creating small depressions,^54–56^ breaking up soil aggregates and filling soil pores,^57–59^ compacting the soil^55,57^ and selectively transporting particles due to size.^59–61^ A combination of these processes could be responsible for the decreases in particle number on the soil surface, by creating a mixing process at a microscale which incorporated the particles into the near surface soil layer. This mixing process may also reduce the likelihood of particle transport in surface runoff, by moving the particles from the surface into the soil matrix. Further, once mixed within the soil, particles could be transported by water infiltrating vertically into the soil profile prior to surface runoff, thereby removing the particles from the erodible layer. The faster rates of decrease on the soil surface prior to surface runoff for MPs compared to sand indicates that MPs are more readily mixed with the soil than sand particles, which could potentially limit MP transport from the soil in surface runoff.

### Microplastic Transport in Surface Runoff

Across all treatments, a mean surface runoff rate of 55.4 ± 21.3 mL min^–1^ was delivered from the soil boxes, with no significant differences between treatments (F­(3,92) = 1.28, *p* = 0.29). Runoff rates through time also showed no notable variation across the soil boxes (Figure S6). The mean sediment transport rate across all treatments was 1.7 ± 1.3 g min^–1^ with no significant differences between treatments (χ^2^ (3) = 1.93, *p* = 0.59). The total amount of sediment transported from the plot throughout the entire rainfall simulation showed no substantial variation between particle type (Figure S6).

The concentration of PMMA and PS in surface runoff was higher in the first 10 min following the start of surface runoff, compared to either the sand or LLDPE_L_ particles (Figure 5). The concentration of sand particles transported from the soil boxes in surface runoff remained relatively constant throughout the simulations, whereas all MP particle types showed lower concentrations in surface runoff as the rainfall simulation progressed. The overall number of particles transported in surface runoff differed significantly between particle type (F­(3,12) = 12.72, *p* = 0.005). Posthoc testing showed that the number of PMMA, PS and sand particles transported in surface runoff were not significantly different at 910 ± 203, 1082 ± 162 and 1159 ± 192, respectively, while the total number of LLDPE_L_ particles collected in surface runoff was significantly lower at 473 ± 118 (*p* < 0.02). The lower number of LLDPE_L_ particles transported in surface runoff compared to the other MPs is likely due to a combination of disparities in recovery rates and specific characteristics of the MPs. More detailed discussion of these issues is provided later in the “comparing transport pathways between different microplastics” section.

**Figure 5:**
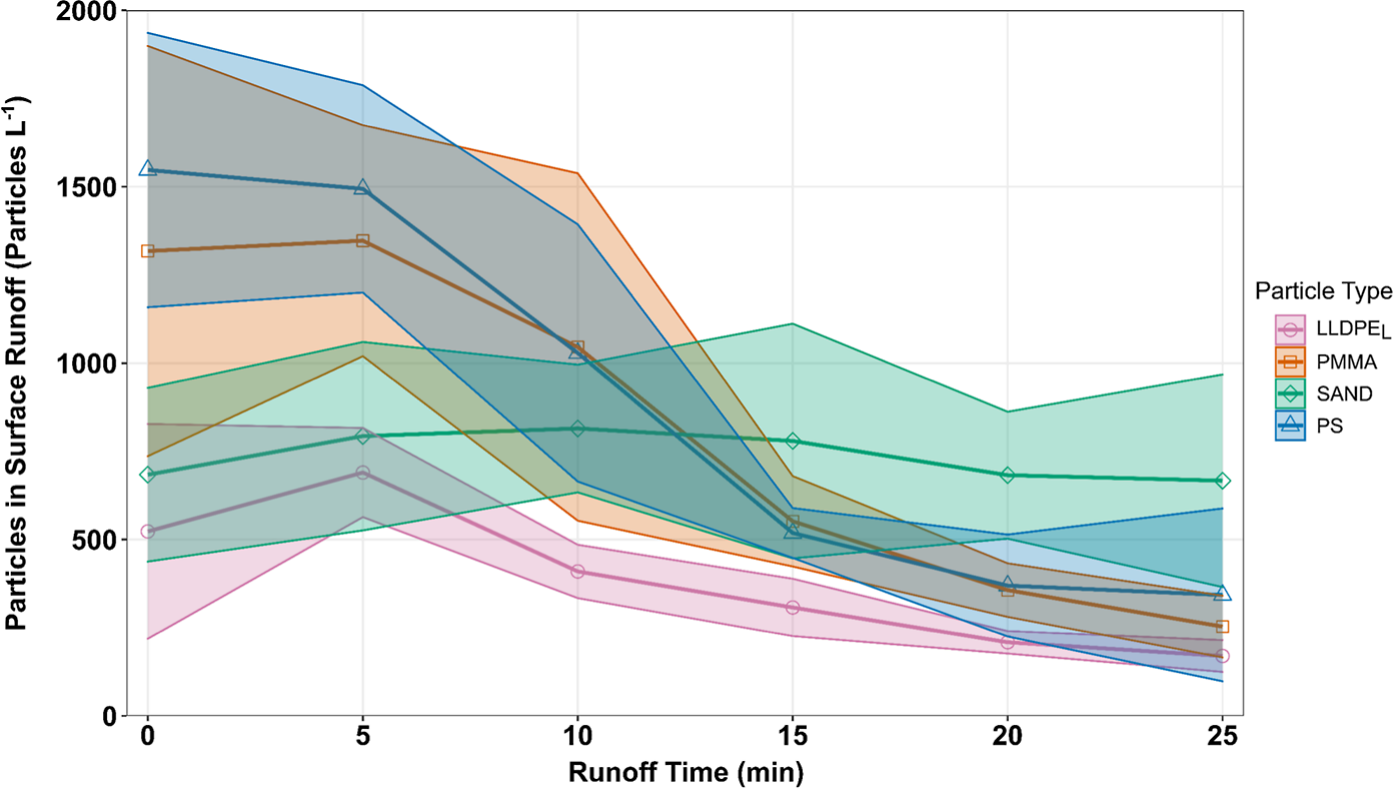
Number of particles per liter of surface runoff through time. Time 0 marks the commencement of surface runoff. Lines represent the mean number of particles while the shaded regions mark ± one standard deviation. LLDPE_L_; PMMA; PS; and SAND represents linear low-density polyethylene size large, poly­(methyl methacrylate), polystyrene and sand particles, respectively.

Once surface runoff commenced, sand particles displayed a significantly slower decrease in particle number at the soil surface as compared to the MPs (Figure 3B). We believe that this reflects higher rates of MP loss from the soil boxes compared to loss of sand particles, due to the combination of: (i) a flush of MPs delivered from the soil boxes in the first 10 min of surface runoff which was not matched by the sand particle treatment in this time period (Figure 5); (ii) preferential splash erosion of MPs compared to sand particles (Figure 4). Rehm et al.^28^ also noted similar patterns of high MP transport in the early stages of surface runoff which subsequently declined dramatically due to an exhaustion of MPs in the erodible soil layer.

### Retention and Transport into the Soil Profile

Based on data from soil cores collected following the end of each rainfall simulation, sand particles were preferentially retained in the soil, at 3432 ± 1434 particles, compared to all MP types which remained below 1600 particles (Figure 6). A Kruskal–Wallis test indicated significant differences in the number of particles retained in the soil among different particle types χ^2^ (3) = 11.71, *p* = 0.008, though posthoc testing did not show statistically significant differences between any pairs of particle types (*p* > 0.17). Across all particle types, rarely did any particle penetrate below the first centimeter of the soil profile (Figure S7 and Table S3), though this is likely due to limited pore space in repacked soil boxes. Under field conditions, where pore structure and connectivity are more developed, MPs are likely to have enhanced vertical movement in the soil. Other research has demonstrated that MPs can be relatively mobile vertically within the soil profile, migrating to depths greater than 10 cm.^52,53,62–65^

**Figure 6:**
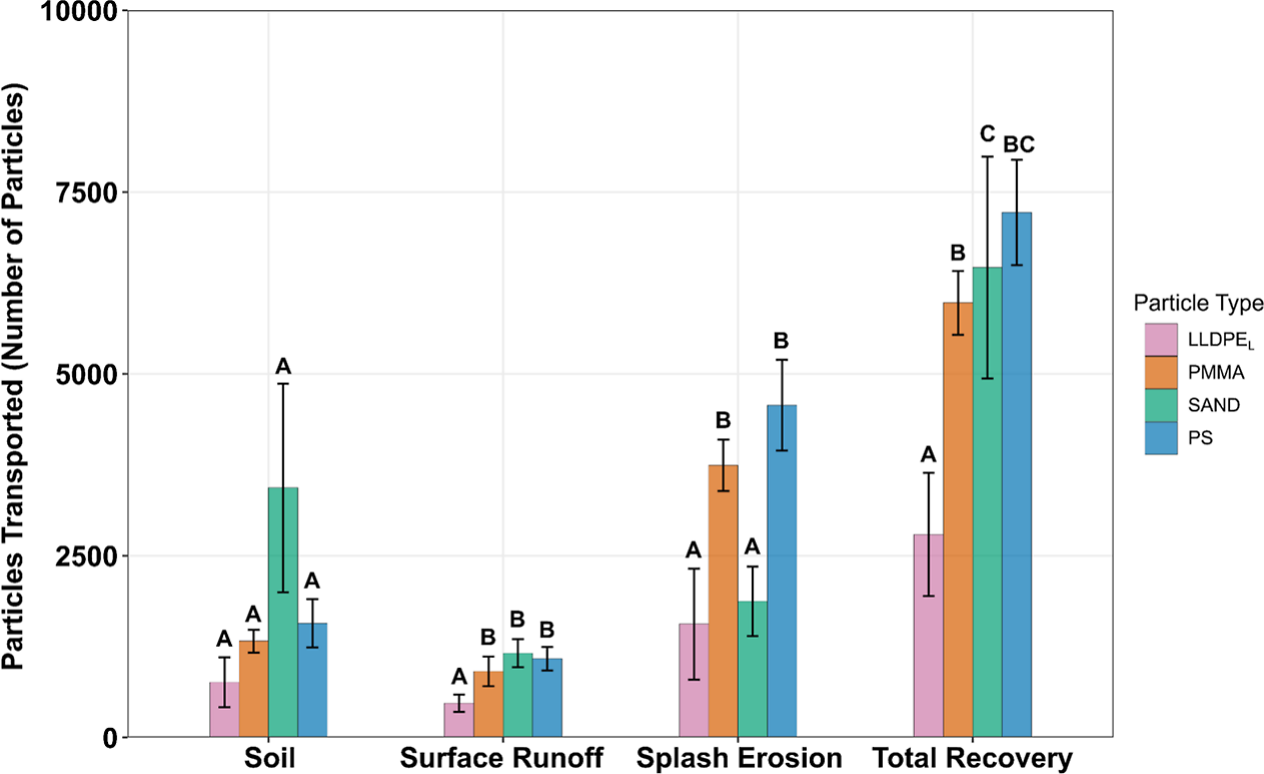
Particles found in each transportation compartment measured. Error bars represent ±1 standard deviation. Letters indicate significant differences between particle types in each transport pathway. LLDPE_L_; PMMA; PS; and SAND represents linear low-density polyethylene size large, poly­(methyl methacrylate), polystyrene and sand particles, respectively.

### Microplastic Transport via Splash Erosion

The estimated number of particles transported via splash erosion to a 1 m circular radius around the soil box was a function of particle type (F­(3,12) = 25.36, *p* < 0.0001), with posthoc tests showing a greater number of PMMA and PS particles, 3742 ± 353 and 4569 ± 625 respectively, being transported outside of the soil boxes, as compared to only 1874 ± 481 sand particles and 1561 ± 765 LLDPE_L_ particles (*p* < 0.003). Particle type had no significant effect on the distance from the soil box that particles were transported via splash erosion up to 1 m (F­(3,12) = 0.591, *p* = 0.63).

After surface runoff began, there was an increase in the rate of MP transport from the soil boxes through splash erosion, compared to the rate of sand particle transport via this mechanism (Figure 4). This observation is consistent with past research on splash erosion mechanics which shows that thin films of water on the surface of a soil can increase the number of particles transported via splash erosion, as compared to dry soils.^66–69^ However, the fluorescent sand particle itself did not reflect this pattern in our research. There are two potential explanations for why MPs were more mobile as compared to the sand particle. First, MPs in this research having densities ranging from 0.92 to 1.19 g cm^–3^ and hydrophobic properties (relative to sand particles) are likely to be buoyant in water, thereby needing less kinetic energy to transport them outside of the soil box via splash erosion as compared to sand particles. Second, when sand particles are transported by splash erosion the higher density and hydrophilic properties (relative to the MPs) of the sand results in shorter transport distances once surface runoff commenced i.e. within the boundaries of the soil boxes. The density of soil particles has been shown to be a controlling factor determining whether or not soil particles are transported via splash erosion, or in surface runoff.^36,70^ Similarly, hydrophobic particles have been shown to be more mobile than hydrophilic particles in soil erosion research.^71,72^ Our results are consistent with research investigating the transport of soil particulate organic carbon that has a similar density to plastics, which found soil organic carbon was more likely to be transported via splash erosion as compared to mineral particles.^73^

### Mass Balance

A mass balance was constructed by summing MP and sand particle numbers found in the surface runoff, retained in the soil and transported through splash erosion processes up to a 1 m radius around the soil box (Figure 6). This total particle number was then compared with the initial assumption that 10,000 particles had been spread on the soil surface prior to the start of the rainfall simulations. The PMMA, PS and sand had the highest recovery rates at 5977 ± 437 (59.8%), 7220 ± 726 (72.2%) and 6466 ± 1525 (64.7%) particles, respectively. The LLDPE_L_ had the lowest recovery rate at 2794 ± 847 (27.9%) particles.

Sand and MP particles were observed in nearly equal numbers in surface runoff. Although this result may suggest that MP and sand particles are transported in relatively equal numbers within surface runoff, it is important to note that the particles which were transported outside of the soil box via splash erosion in our experiments would have been entrained in surface runoff under field conditions. Therefore, it is likely that under large-scale field conditions, MPs would have been transported in greater numbers than sand particles in surface runoff.^28^ Combined splash erosion and surface runoff accounted for 57% of PS, 47% of PMMA recovered from experiment compared to 30% of recovered sand particles and 20% of recovered LLDPE_L_.

The discrepancy in the mass balance, where no single particle type achieved 100% recovery, could be attributed to several factors. First, the use of weight-to-particle number ratios may have introduced uncertainties in our estimates of the total number of particles input to the soil surface at the start of the simulations. For example, PS consistently had a slightly higher number of particles detected by the camera on the soil surface at time 0 than the other treatments, which may explain why slightly more PS was recovered than PMMA. Second, not all areas of particle deposition were captured using the measurement approach we developed. For example, it was observed that particles accumulated on a 3 cm vertical edge between the soil surface and the top of each soil box, but as the camera was not able to capture images of the edge these particles were not accounted for. Additionally, it is possible that particles were transported by surface runoff out of the soil box before the first surface runoff sample was collected. Lastly, in the case of LLDPE_L_ limitations of the image-based particle detection method as discussed above likely contributed to a lower recovery rate as compared to all other particle types. Therefore, the significant differences observed between the LLDPE_L_ and other particle types should be interpreted with caution as they likely stem from methodological limitations rather than intrinsic disparities in transport behavior. However, the inclusion of the LLDPE_L_ remains informative, particularly given that its reduced recovery appears to be consistent across all transport pathways, suggesting no systematic bias in its behavior.

### Comparing Transport Pathways between Different Microplastics

In this experiment, PS and PMMA demonstrated nearly identical transport rates and dominant transport pathways. These particles were very similar in physical characteristics, sharing the same morphology and size, though they possess slightly different densities. There were several significant differences in transport pathway (Figure 6), and number of particles detected on the surface of the soil throughout the rainfall simulation (Figure 2), when comparing the PS and PMMA particles to the LLDPE_L_ particles. The lower recovery rate of LLDPE_L_ in the mass balance and the difficulty in distinguishing these particles from the background soil, likely contributed to differences observed in the transport pathways and rates of movement over the soil surface compared to PS and PMMA. However, the larger size, greater plasticity, and flake-like morphology of LLDPE_L_ compared to PS and PMMA could have also played a role in the faster rates of decline from the soil surface prior to surface runoff (Figure 3A) and the number of particles in each transport pathway.^26,50^ With a higher plasticity, LLDPE_L_ may “bend” with the impact of raindrops rather than being transported short distances (on the mm-cm scale within the soil box), resulting in more extensive mixing of LLDPE_L_ with the near-surface soil compared to either the PMMA and PS particles. Additionally, research investigating MP transport in surface runoff has generally shown that MP particles with larger surface areas, such as LLDPE_L_ in our research, are less likely to be mobilized in surface runoff.^26,27^

Understanding the influence of morphology and polymer type on MP mobilization and transport is complex, because morphology is often at least partly related to polymer type. Additionally, polymer type has several characteristics, aside from density, which could influence particle movement i.e., plasticity, surface charge, and hydrophobicity. The lack of significant differences in transport between PMMA and PS MPs in our research challenges the assumption that marginal differences between MPs, such as density, make a significant difference to transport processes in rainfall-induced erosion.^74,75^

### Limitations

While this study provides important insights into microplastic transport processes, several limitations should be acknowledged. First, the overarching aim of this experiment was to provide the first direct comparison of transport processes for MPs and a natural soil particle and identify differences in their transportation mechanisms. This necessitated a controlled laboratory experiment. The use of small soil boxes in erosion experiments is valuable for gaining detailed understanding of erosion processes^76^ although it is well documented that results from lab-based experiments are not always directly translatable to the field or catchment scales.^77^ Specifically, small scale erosion experiments often have higher amounts of runoff and sediment transport compared to observations under natural conditions.^78,79^ Additionally, the lack of aggregation with soil particles and lack of vegetation in our experiments may lead to increased transport of MPs and sand particles compared to the field or catchment scale. However, the results from small-scale experiments are valuable as they provide mechanistic insights into particle movement which can be used to develop and adapt high-resolution, processed based transport models. Second, MPs currently represents an extremely broad term which includes plastics of all polymer types, morphologies, degrees of degradation, and sizes from 1 μm to 5 mm.^80^ Consequently, MP transport rates are expected to vary based on these physicochemical characteristics.^26–28,50^ Although not every possible size, morphology, or polymer type was examined in our experiment, the results we report reveal how bulk differences between MPs and soil particles, for example in terms of hydrophobicity or density, influence transport. Further, while the MPs used in our experiments did not undergo environmental aging, we believe that the results we report provide important insights into the transport behavior of MPs that are newly introduced into soils, or rapidly generated from macroplastics via mechanical fragmentation such as via tillage^81–84^ or abrasion. Overall, there is a pressing need for more experimental research investigating the processes controlling MP transport during erosion events and how the transport of MPs differs from natural soil particles. Future research should aim to understand how chemical differences (e.g., hydrophobicity, plasticity, surface charge) between soil particles and MPs influence transport processes in erosion events, as well as consider how changes in the physical and chemical properties of MPs during aging influence the transport behavior of these particles within soils.

### Environmental Implications

Our research provides new insights into the transport of MPs relative to mineral soil particles during rainfall-induced erosion events by presenting the first direct, side-by-side comparison of mechanisms governing their transport. The combined impact of splash erosion and surface runoff demonstrates the preferential mobility of MPs in erosion processes compared to mineral soil particles. Additionally, MPs exhibiting an initial “flush” from the soil indicates that even in short duration heavy rainstorms a substantial amount of MPs could be transported from the soil. While several strides have been made in understanding MP mobility in erosion events, our research showed that small-scale experiments which do not account for MP transport through splash erosion processes may misrepresent dominant transport mechanisms or total MP fluxes from the soil. Despite the highly mobile nature of MPs in surface runoff, our research also highlights the potential role of splash erosion processes in facilitating the vertical transport of MPs. Through the “real time” tracking of MPs on the soil surface, we uncovered that prior to surface runoff, within the first 10 min of the rainfall simulations, 70–80% of MPs disappeared from the soil surface, with only 3–5% of this figure attributed to MP transport out of the soil boxes. The remaining ∼65–75% loss of MPs was attributed to the mixing of MP particles with the soil matrix, which could serve as a gateway for further vertical transport in agricultural soils. While we report little evidence of MP or sand particles moving below 1 cm depth in the soil profile during our experiments—likely due to limited pore space in repacked soil boxes—this process may act differently in agricultural soils under field conditions, in which pore structure and connectivity may allow for enhanced movement of MPs vertically within the soil profile. Under these conditions, the initial burial of MPs could facilitate further vertical transport by infiltrating water, leading to additional MP retention in the soil.

Overall, our research highlights the critical role of erosion as a transport mechanism for MPs entering aquatic ecosystems. Mitigating MP transport to aquatic environments may be achieved through soil erosion control measures such as cover crops,^27^ vegetative buffer strips and restoring riparian areas. Implementing strategies such as these can help reduce surface runoff, stabilize soil and ultimately limit MP mobilization from the terrestrial environment to aquatic ecosystems.

## Supplementary Material



## Data Availability

Data is available via Zenodo: 10.5281/zenodo.14354938.

## Notes

Text describing; experimental setup, preparation of the fluorescent LLDPE particles, recovery of the LLDPE_S_ particle, weight-to-particle number ratio calculation, camera settings, images analysis methodology and evaluation; Tables showing the thresholding values for particle identification, performance evaluation of the particle identification, the vertical migration of particles into the soil profile; Figures showing laboratory setup, soil characteristics and sediment delivery, microscope images of the particles, detection of particles on blank soil surfaces, water runoff and sediment delivery, vertical movement of particles (PDF)
