# Supplementary material for: The Transport of Microplastics from Soil in Response to Surface Runoff and Splash Erosion
Source: Environ Sci Technol. 2025 Jul 2;59(27):14063–74. doi: 10.1021/acs.est.5c04795 (PMC12269087; doi:10.1021/acs.est.5c04795)
Supplement: Supplementary file 1 [file es5c04795_si_001.pdf]

Supplementary Information

The transport of microplastics from soil in response to surface runoff and splash erosion

Emilee Severe[1*], Ben W. J. Surridge[1], Peter Fiener[2], Michael P. Coogan[3], Rachel H. Platel[3], Mike R. James[1], John Quinton[1]

1. Lancaster Environment Centre, Lancaster University, Lancaster, LA1 4YQ, United Kingdom
2. Institute of Geography, University of Augsburg, Alter Postweg 118, 86159 Augsburg, Germany
3. Department of Chemistry, Lancaster University, Lancaster, LA1 4YB, United Kingdom

*Corresponding Author

Email address: e.severe@lancaster.ac.uk

This supplementary material is a 13 page document and includes 3 tables, 7 figures, descriptions and references.

1.1 Experimental Set-up

Soil used in this research was a loamy sand topsoil with the following particle size range distribution: 7.8 ±1.7% clay; 7.6 ± 0.4 % silt; 84.7 ±1.9 % sand. Organic matter content of the soil was 3%. The loamy sand soil was selected as it is a common soil texture used in agriculture due to its properties which facilitate good drainage and nutrient retention. Several studies have identified plastic residues from soils of this texture or similar textures[1–3]. Further, soil was sourced from Norfolk County, UK where plastic mulch is known to be used for vegetable production [4].

Soil boxes were packed layer by layer first by adding ~3.6 kg of soil (which created ~2.2 cm layer of soil) then evenly spreading 500 mL of tap water on the surface with a watering can. This process was repeated until the soil reached a depth of 11 cm after which the soil box was covered with a lid and left for 24 hours to allow for homogenization of the water in the soil. This resulted in a bulk density of 1.3 g cm^{-3} (Fig S2 D) and a volumetric water content of 19%. Soil boxes were set at a 10-degree slope to reflect realistic environmental topography whilst ensuring runoff generation in a practical time frame.

Rainfall was produced from a gravity-fed rainfall simulator (Fig. S1) set at 50 mm hr^{-1} with a Christiansen's coefficient of 84.5% [5]. Tap water was used as a water supply for the rainfall simulator. The rainfall rate from this simulator is known to decline after a few hours of operation[6], therefore the rainfall rate was measured before each rainfall simulation and no more than two rainfall simulations were conducted in one day. Prior to each rainfall simulation the rainfall rate was measured twice to ensure consistent rainfall rates across all replicates and treatment. Overall, a mean rainfall rate of 49 ± 2 mm h^{-1} was recorded (Fig. S2 C). The simulator was mounted 3.27 m above the ground and produced raindrops ranging 0.9 mm- 3.5 mm with the mean raindrop size being 1.8 mm resulting in a kinetic energy of approximately 14.59 J m^{-2} mm^{-1}.

1.2 Preparation of Fluorescent LLDPE Particles

To a solution of rhodamine B chloride (Sigma Aldrich) (25 mg, 0.052 mmol) in acetonitrile (2.5 mL) in a 10 mL round bottom flask equipped with a stirrer bar was added silver (I) trifluoromethane sulphonate (14 mg, 0.055 mmol) in acetonitrile (2.5 mL). The flask was flushed with nitrogen, sealed with a greased stopper loosely attached with rubber bands and the mixture stirred at ambient temperature for 10 minutes. The resulting thick suspension was filtered through celite, washed with 2 x 1 mL acetonitrile and concentrated under vacuum to give a very dark red solid that was used without further purification in the next step.

To a 500 mL round bottomed flask equipped with a large stirrer bar was added 20 g of linear low density PE pellets (Sigma Aldrich) and 250 mL of toluene. The mixture was heated in an oil bath set to 100 C with stirring, initially magnetic and then, as the mixture thickened, manual with a large spatula, and periodic addition of portions of toluene to maintain the volume as it was absorbed into the mixture. Once the beads were all uniformly swollen (ca. 30 minutes) 0.4 mL of a 0.01M solution of Rhodamine B Trifluoromethanesulphonate salt in acetonitrile was added dropwise with rapid stirring to disperse the highly coloured areas left after the immediate evaporation of the acetonitrile (CAUTION – over-rapid addition can lead to violent evaporation). Stirring was continued at 100 C until the swollen beads were uniformly coloured, and then the oil bath temperature was increased to 140 C and manual

stirring continued until the mixture was homogenous (ca. 1 hour). Residual toluene was evaporated under a stream of compressed air, and then the mixture was allowed to cool to room temperature. The polymer was cut into 0.5-1 cm cubes and dried in a vacuum oven at 70 C overnight or to a constant mass.

1.3 $LLDPE_S$ Recovery

$LLDPE_S$ particle (sized 250-355 µm) showed poor recovery in images of the soil surface, splash mat, surface runoff and soil samples, with <1% of the particles inputted recovered. This poor recovery led us to exclude the $LLDPE_S$ particle from the results. Both the $LLDPE_L$ and $LLDPE_S$ particles were dyed using Rhodamine B. Rhodamine B's excitation and emission peak wavelength is 546 nm and 567 nm, respectively. In order to fluoresce the MP and sand particles with multiple peak excitation wavelengths, a UV light with a peak emission of 365 nm was used. The commercially dyed MPs and sand particles were easily detectable, the LLDPE particles proved more difficult. As $LLDPE_L$ particles had larger amounts of dye due to its larger size (500-600 µm), they were more readily detected than the $LLDPE_S$ particles.

1.4 Weight-to-Particle Number Ratio Calculation

In order to ensure a similar number of MPs and sand particles in each soil box, a weight-to-particle number ratio was calculated for each MP polymer type and for the sand particle. Known weights of MPs and sand particles (ranging from 0.2- 2.5 mg) were manually counted in 3-5 replicates. For each replicate, the weight per particle was determined, and the average was calculated across all replicates to obtain the mean particle weight. This mean particle weight was then multiplied by 10,000 to determine the total weight of particles needed per soil box to achieve a distribution of 10,000 particles on the soil surface.

1.5 Camera Settings

The camera capturing the surface of the soil box was a Canon EOS 850D with a 50 mm prime lens. Images were captured with the following settings: f/9.0, exposure 1 sec., ISO 320, RAW+ JPEG. An intervalometer (Neewer RS-60E3) automatically captured the images every 10 seconds. The resolution of the images were 6000 x 4000 pixels. Camera was set at the base of the soil box approximately 50 cm away from the soil box and at a height of 140 cm. The camera used to capture the splashed particles was a Canon 500D with a 50 mm prime lens. Images were captured with the following settings: f/9.0, exposure 1 sec., ISO 200, RAW+JPEG. An intervalometer (Neewer RS-60E3) automatically captured the images every 60 seconds. Following methods developed by [7], both cameras capturing the real time movement were first autofocused with artificial lights on then switched to manual focus and the focus ring was manually secured to prevent focus drift due to shutter vibrations.

At the conclusion of the experiment the total area of the 1 m^2 splash mat was photographed. The EOS 850D with a 50 mm prime lens was used to photograph the mat. The 1m^2 mat was divided into quarters using 50 cm^2 metal quadrats. Each quadrat was photographed separately with the camera at a height of approximately 210 cm in order to capture the whole sectioned area (Fig. S4). Both of the UV lamps were repositioned to fully illuminate each section as it was photographed.

1.6 Color Thresholding

Using the Color Thresholding tool in image J microplastics and sand particles were identified and then counted. The color thresholding converts images from RGB to binary images by segmenting pixels to set values. The HSB color space was used in the segmentation of the image as it is robust against changes in illumination [8]. Different thresholds were used for the surface of the soil and the splash mat (Table S1). As the rainfall simulation progressed through time, the surface runoff reflected the light of the UV lamp, so thresholds which excluded pixels of high brightness values and retained high saturation values were used.

Table S1: Threshold ranges for particle detection as used in image J.

Soil Surface				Splash Mat			
Particle	Hue	Saturation	Brightness	Particle	Hue	Saturation	Brightness
$LLDPE_L$	20-225 "pass" or band-reject	85-255	45-255	$LLDPE_L$	20-225 "pass" or band-reject	85-255	35-255
$LLDPE_S$	20-195 "pass" or band-reject	85-255	40-255	$LLDPE_S$	20-225 "pass" or band-reject	70-255	30-255
SAND	50-130	40-255	65-255	SAND	50-130	85-255	45-255
PMMA	0-55	30-255	150-255	PMMA	0-55	30-255	85-255
PS	0-55	55-255	150-255	PS	0-55	60-255	70-255

1.7 Performance Evaluation

Performance of the image analysis was evaluated by calculating the recall, precision and f-score for each particle type as shown in Table S2. Recall, precision and f-score are calculated on a scale of 0 to 1, with 1 reflecting the highest performance . Recall describes the percentage of particles detected which were correctly classified (true positive/(true positive +false negative)). Precision describes the proportion of the positives detected that were correctly classified as particles (true positive /(true positive +false positive)). F-score considers both the precision and the recall to measure the proportion of particles which were correctly classified in the images (2*((precision*recall)/(precision +recall)).

Table S2. Table showing the correct and incorrect identification of fluorescent particles in images.

Soil Surface				Splash Mat			
Particle	Recall	Precision	F-score	Particle	Recall	Precision	F-score
$LLDPE_L$	0.887	0.882	0.885	$LLDPE_L$	0.885	0.746	0.810
SAND	0.838	0.978	0.903	SAND	0.973	0.971	0.972
PMMA	0.882	0.979	0.928	PMMA	0.994	0.995	0.994
PS	0.792	0.997	0.883	PS	0.993	0.994	0.994

1.8 Effectiveness of the image-based detection method

The dynamic nature of the soil surface, including variations in surface roughness and the onset of surface runoff, and the movement of particles during the rainfall simulations, makes it challenging to detect particles on the soil surface using image-based detection methods. To

ensure patterns of particle movement on the soil surface observed in this research can be attributed to transport processes and not simply artifacts of the detection method, we used blank soil boxes without any particles added to the soil as well correlated the number of particles observed on the surface with particles identified in each transport pathway.

Fig. S5 shows the number of particles detected on the surface of the blank soil boxes for each particle type. The number of particles detected on the blank soil surface never exceeded 800 particles of any particle type. The number of particles detected on replicate box 1, was much higher for the $LLDPE_L$, $LLDPE_S$ and PMMA particles compared to all the other replicates. This was not reflected for the PS and sand particles. When replicate box 1 is excluded from the data the number of particles does not exceed 400. There was a pattern of more particles detected on the images of the surface of the blank soil boxes in the latter end of the simulations. The number of particles detected on the splash mat from the blank soil boxes was relatively low with $LLDPE_L$, PMMA, PS and sand particles consistently detecting less than 25 particles. $LLDPE_S$ on the other hand constantly detected more than 25 particles over the course of the simulation.

Correlating the patterns of decrease on the soil surface (Fig. 2) with the number of particles identified in each transport pathway gives evidence that images of the soil surface reflect transport patterns and not artefacts of the detection method. For example, the largest decrease in particle number on the soil surface occurred in the first 10 minutes of the rainfall simulation (Fig. 2), and visual inspection of the respective images showed that only 3 of the 16 soil boxes had evidence of a layer of water on the surface at this time. Therefore, interference in the detection method due to water on the soil surface is believed to be unlikely. Likewise, after surface runoff began, the number of particles transported from the soil boxes in surface runoff and splash erosion approximately accounts for the decrease in particle number observed on the soil surface (Figs 2 & 4.) However, the $LLDPE_L$ had the weakest fluorescence signal than all other particles, as the optimal wavelength to excite the Rhodamine dye (~500 nm) was not used in the experiment, but rather a UV light with a peak wavelength of 365 nm. For this reason, it was difficult to distinguish $LLDPE_L$ from soil in the images, which resulted in a lower recovery from the images compared to the other particle types.

Table S3. Table showing the mean and standard deviation of microplastics found at each soil layer. Surface particles were counted once sampling core was in the soil then ultimately subtracted from particles found in the 0-1 cm depth class. MP and sand particles detected in soil samples were extrapolated to the area of the soil box. $LLDPE_L$*; PMMA; PS; and SAND represents linear low-density polyethylene size large, polymethyl methacrylate, polystyrene and sand particles, respectively.*

Soil Sample Depth	$LLDPE_L$	PMMA	SAND	PS
Surface	281 ± 247	441 ± 336	1414 ± 540	418 ± 439
0-1 cm	475 ± 327	878 ± 442	2166 ± 1417	1129 ± 522
1-2 cm	3 ± 14	3 ± 14	24 ± 40	7 ±19
2-3 cm	0 ± 0	0 ± 0	0 ± 0	7 ± 19
3-4 cm	0 ± 0	3 ± 14	17 ± 33	17 ± 35

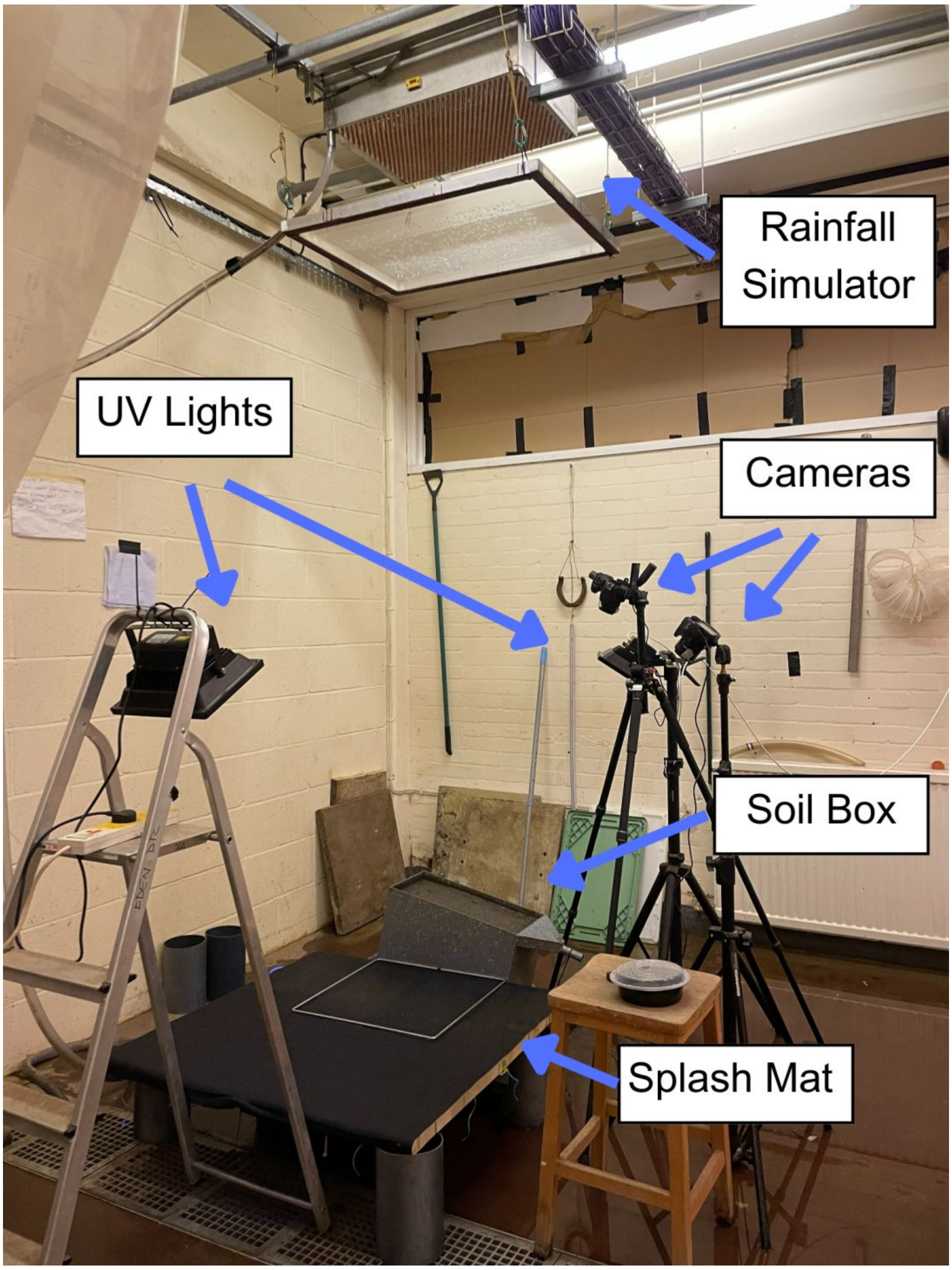

Fig. S1: Laboratory set-up for simulation experiment.

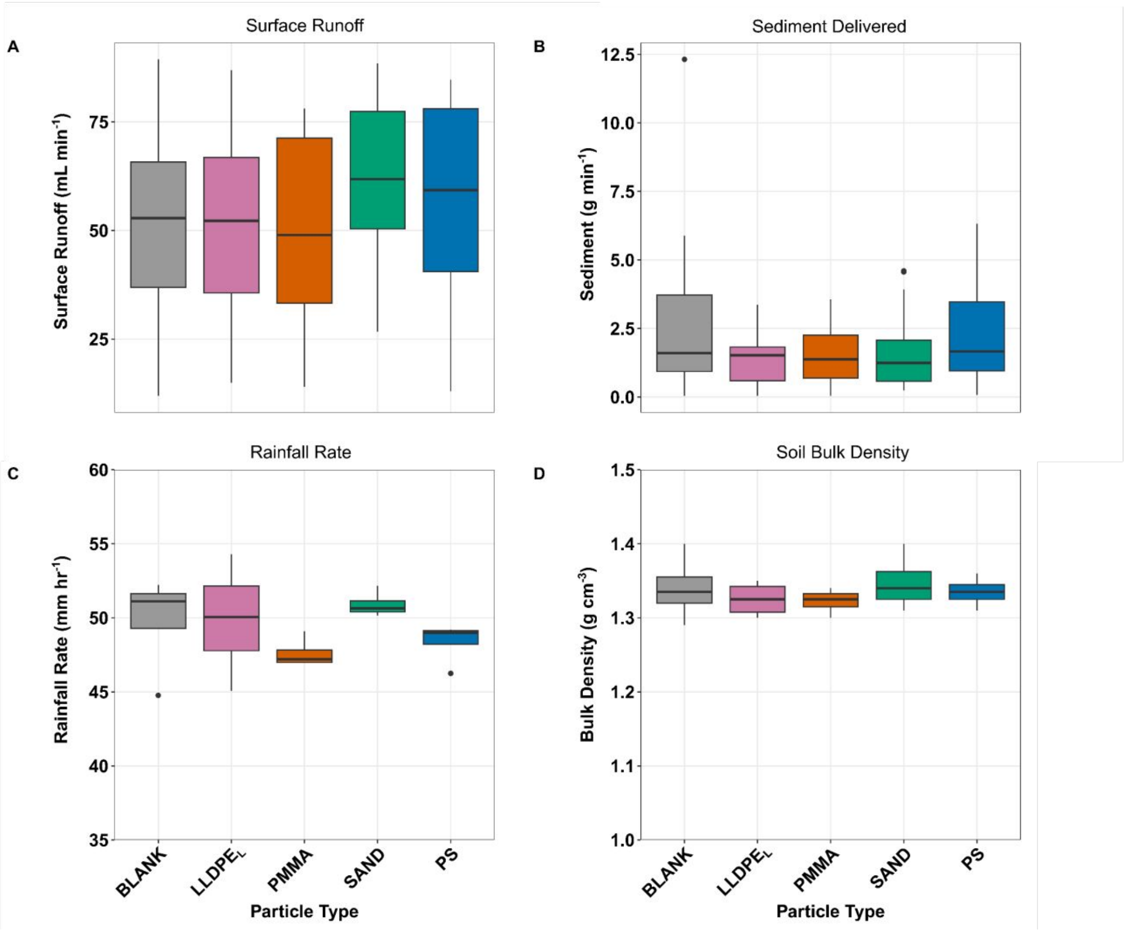

Fig. S2: Graphs showing factors of the experiment. Panel A shows the amount of runoff delivered from each plot. Panel B shows the amount of sediment delivered from each plot. Panel C shows the rate of rainfall in mm hr^{-1}. Panel D shows the bulk density of the soil in the soil boxes. Across all treatments, no extreme variations were detected. LLDPE$_L$; PMMA; PS; SAND; and BLANK represents linear low-density polyethylene size large, polymethyl methacrylate, polystyrene, sand particles, and soil with no particles added respectively.

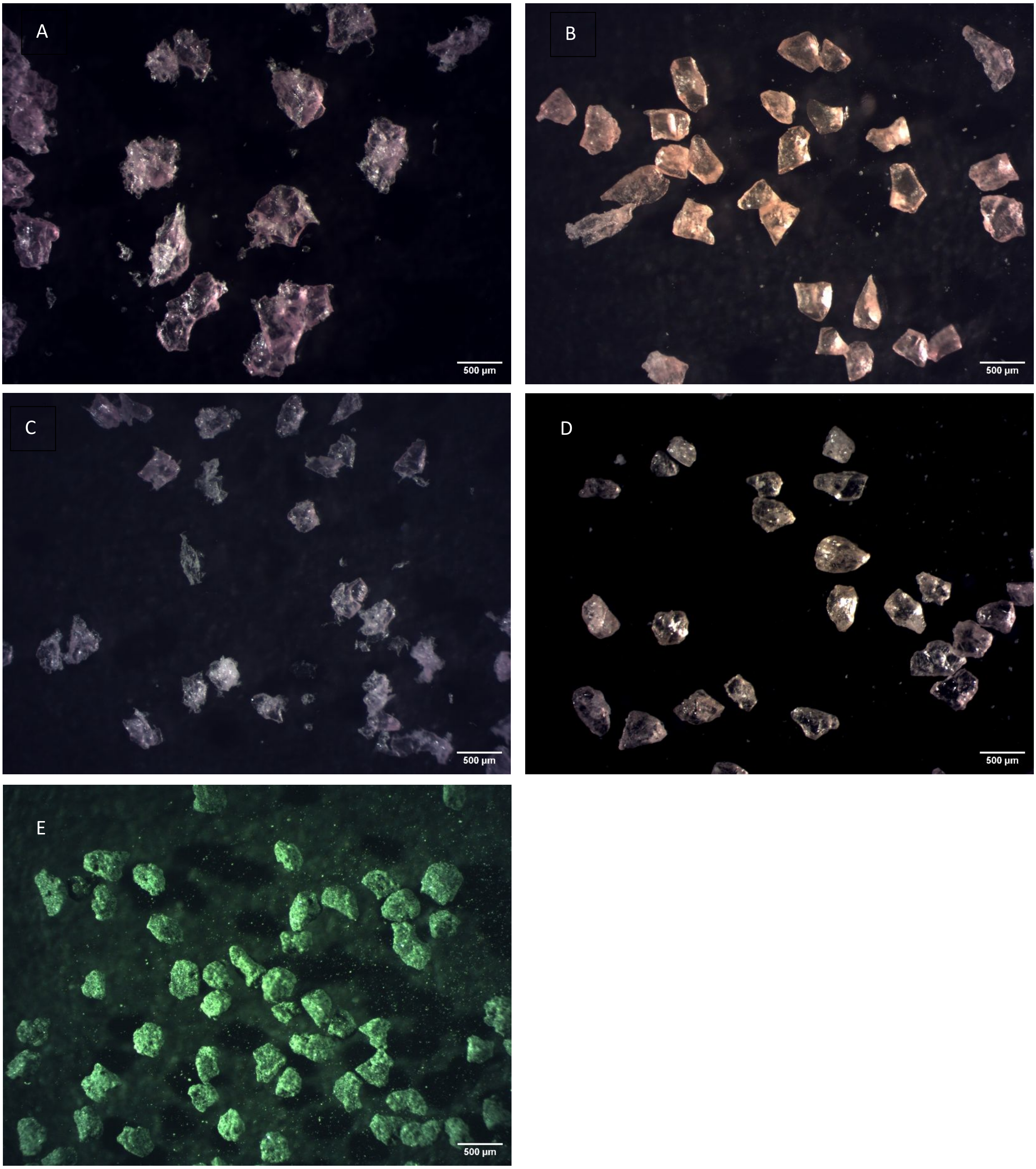

Fig. S3: Images of the particles used in this experiment. Where image A represents linear low-density polyethylene large MP ($LLDPE_L$), B represents polymethyl methacrylate MP (PMMA), C represents linear low-density polyethylene small MP ($LLDPE_S$), D represents polystyrene MP (PS), and E represents the sand particle.

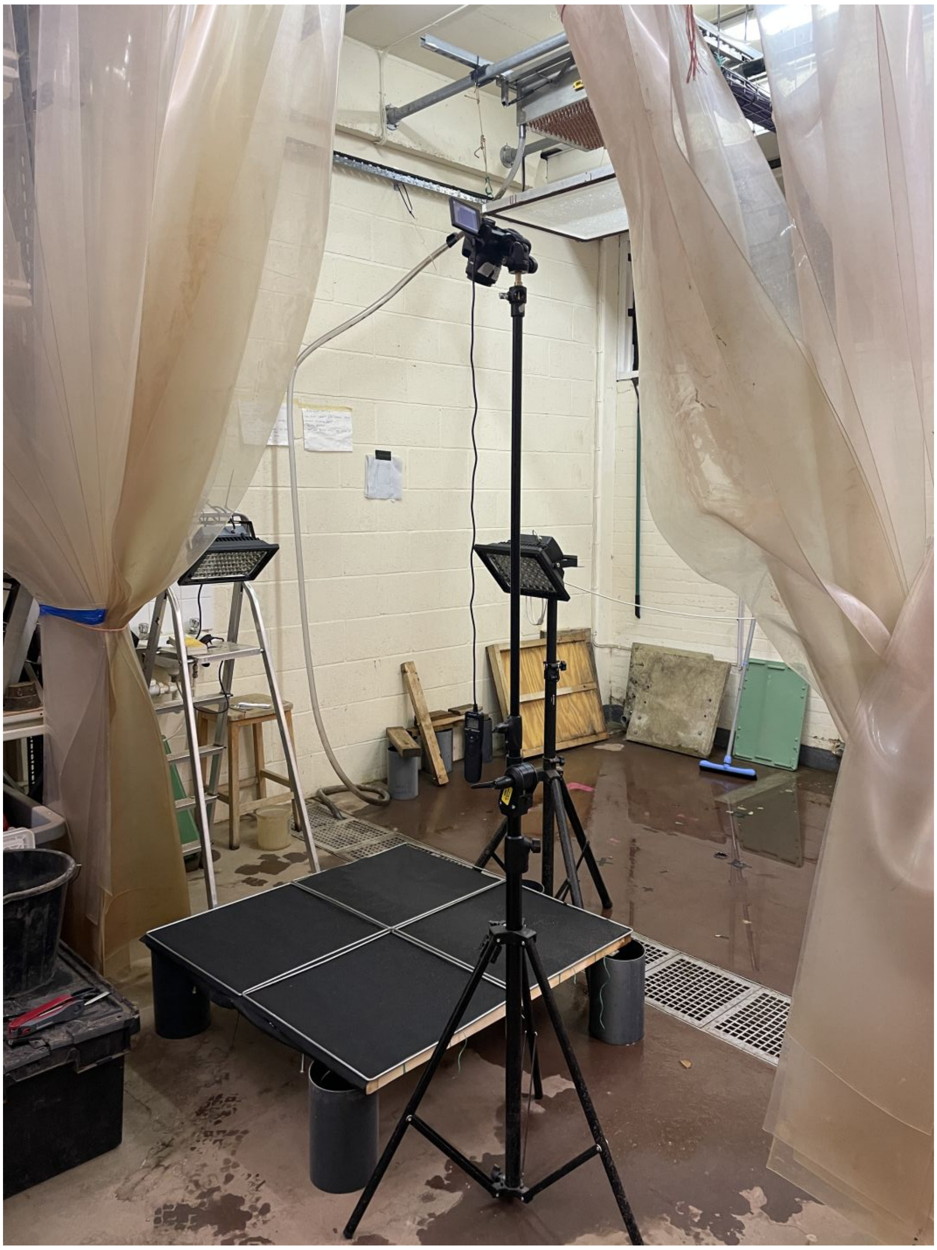

Fig. S4: Camera and light set-up to photograph the 1m² splash mat at the conclusion of a rainfall simulation.

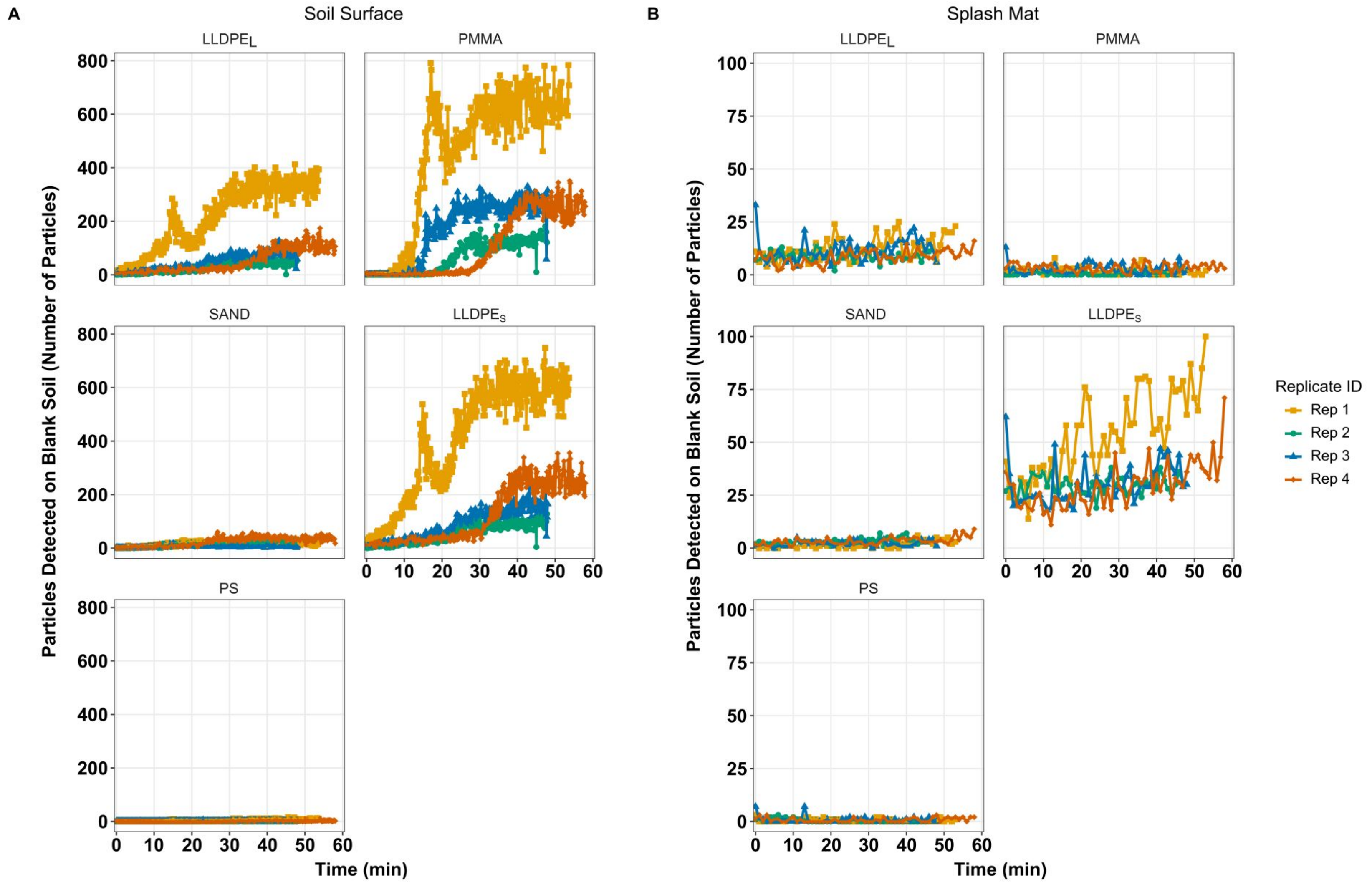

Fig. S5: Figures showing the number of particles detected in the image analysis process on blank soil throughout the rainfall simulation. Panel A shows particles on the surface of the soil box and panel B shows the particles on the splash mat. Each line represents a different replicate. LLDPE$_L$; PMMA; PS; SAND; and LLDPE$_S$ represents linear low-density polyethylene large size, polymethyl methacrylate, polystyrene, sand particles, and linear low-density polyethylene small size respectively.

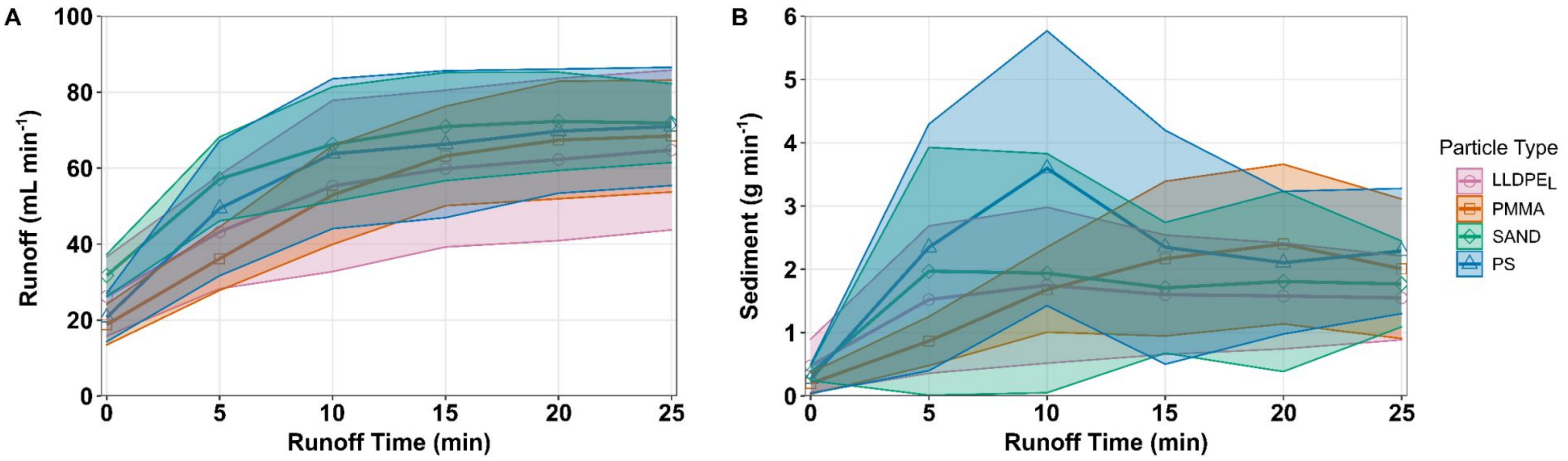

Fig. S6: Surface runoff (A) and sediment flux (B) during the rainfall simulations. Lines and points represent the mean while the shaded regions mark ± one standard deviation. Data is separated by soil boxes containing different particle types. $LLDPE_L$; PMMA; PS; and SAND represents linear low-density polyethylene size large, polymethyl methacrylate, polystyrene and sand particles, respectively

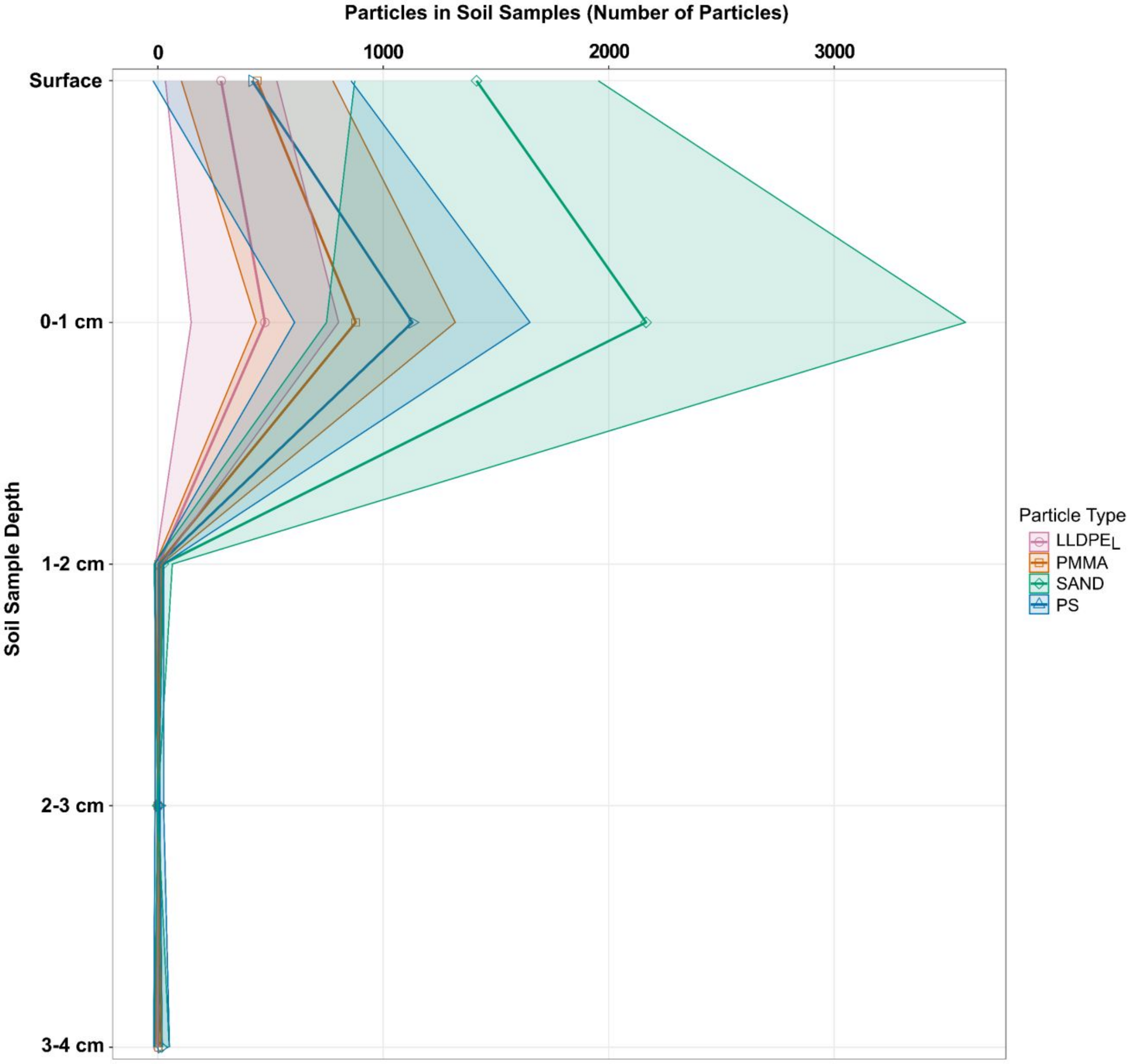

Fig. S7: Vertical movement of MPs through the soil profile to a 4 cm depth. Dark lines represent the mean while the shaded regions mark ± one standard deviation. Surface particles were counted once sampling core was in the soil then ultimately subtracted from particles found in the 0-1 cm depth class. Rarely were any particles found in the soil below a 1 cm depth. LLDPE$_L$; PMMA; PS; and SAND represents linear low-density polyethylene size large, polymethyl methacrylate, polystyrene and sand particles, respectively.

References

(1) Cao, L.; Wu, D.; Liu, P.; Hu, W.; Xu, L.; Sun, Y.; Wu, Q.; Tian, K.; Huang, B.; Yoon, S. J.; Kwon, B.-O.; Khim, J. S. Occurrence, Distribution and Affecting Factors of Microplastics in Agricultural Soils along the Lower Reaches of Yangtze River, China. *Sci. Total Environ.* **2021**, *794*, 148694. https://doi.org/10.1016/j.scitotenv.2021.148694.

(2) Yu, L.; Zhang, J.; Liu, Y.; Chen, L.; Tao, S.; Liu, W. Distribution Characteristics of Microplastics in Agricultural Soils from the Largest Vegetable Production Base in China. *Sci. Total Environ.* **2021**, *756*, 143860. https://doi.org/10.1016/j.scitotenv.2020.143860.

(3) Weber, C. J.; Opp, C. Spatial Patterns of Mesoplastics and Coarse Microplastics in Floodplain Soils as Resulting from Land Use and Fluvial Processes. *Environ. Pollut.* **2020**, *267*, 115390. https://doi.org/10.1016/j.envpol.2020.115390.
(4) Cusworth, S. J.; Davies, W. J.; McAinsh, M. R.; Stevens, C. J. A Nationwide Assessment of Microplastic Abundance in Agricultural Soils: The Influence of Plastic Crop Covers within the United Kingdom. *PLANTS PEOPLE PLANET* **2024**, *6* (2), 304–314. https://doi.org/10.1002/ppp3.10430.
(5) Christiansen, J. E. *Irrigation by Sprinkling*; University of California Berkeley, 1942; Vol. 4.
(6) Pryce, O. Development of Environmental Tracers for Sediments and Phosphorus. PhD Thesis, Lancaster University (United Kingdom), 2011. https://core.ac.uk/download/pdf/196591388.pdf.
(7) Hardy, R. A.; Pates, J. M.; Quinton, J. N.; Coogan, M. P. A Novel Fluorescent Tracer for Real-Time Tracing of Clay Transport over Soil Surfaces. *CATENA* **2016**, *141*, 39–45. https://doi.org/10.1016/j.catena.2016.02.011.
(8) Gonzalez, R. C.; Woods, R. E. *Digital Image Processing*, 3rd ed.; Pearson/Prentice Hall: Upper Saddle River, N.J, 2008.
